# An Improved Method and Device for Nucleic Acid Isolation Using a High-Salt Gel Electroelution Trap

**DOI:** 10.1021/acs.analchem.4c03720

**Published:** 2024-09-17

**Authors:** Ruslan Kalendar, Konstantin I. Ivanov, Ilyas Akhmetollayev, Ulykbek Kairov, Olga Samuilova, Timo Burster, Andrey A. Zamyatnin

**Affiliations:** † National Laboratory Astana, Nazarbayev University, Kabanbay batyr Ave. 53, Astana, 010000, Kazakhstan; ‡ Department of Microbiology, University of Helsinki, Viikinkaari 9, Helsinki 00014, Finland; § Research Center for Translational Medicine, Sirius University of Science and Technology, Olympic Ave., 1, Sochi 354340, Russian Federation; ∥ National Center for Biotechnology, Kurgalzhynskoye Road, 13/5, Astana 010000, Kazakhstan; ⊥ Department of Biochemistry, Sechenov First Moscow State Medical University, Trubetskaya Str. 8-2, Moscow 119991, Russian Federation; # HSE University, Profsoyuznaya Str. 33-4, Moscow 10100, Russian Federation; ● Faculty of Bioengineering and Bioinformatics, Lomonosov Moscow State University, Leninskie Gory 1-73, Moscow 119234, Russian Federation; □ Belozersky Institute of Physico-Chemical Biology, Lomonosov Moscow State University, Leninskie Gory 1-40, Moscow 119234, Russian Federation; % Department of Biology, School of Sciences and Humanities, Nazarbayev University, Kabanbay Batyr Ave. 53, Astana 010000 Kazakhstan

## Abstract

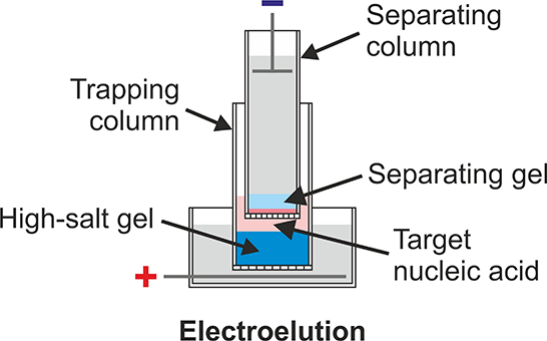

The success of DNA analytical methods, including long-read sequencing, depends on the availability of high-quality, purified DNA. Previously, we developed a method and device for isolating high-molecular-weight (HMW) DNA for long-read sequencing using a high-salt gel electroelution trap. Here, we present an improved version of this method for purifying nucleic acids with high yield and purity from even the most challenging biological samples. The proposed method is a significant improvement over the previously published procedure, offering a simple, fast, and efficient solution for isolating HMW DNA and smaller DNA and RNA molecules. The method utilizes vertical gel electrophoresis in two nested, partially overlapping electrophoretic columns. The upper, smaller-diameter column has a thin layer of agarose gel at the bottom, which separates nucleic acids from impurities, and an electrophoresis buffer on top. After the target nucleic acid has been gel-purified on the upper column, a larger-diameter column with a layer of high-salt gel overlaid with electrophoresis buffer is inserted from below. The purified nucleic acid is then electroeluted into the buffer-filled gap between the separating gel and the high-salt gel, where excess counterions from the high-salt gel slow its migration and cause it to accumulate. The proposed vertical purification system outperforms the previously described horizontal system in terms of ease of use, speed, scalability, and compatibility with high-throughput workflows. Furthermore, the vertical system allows for the sequential purification of several nucleic acid species from the same sample using interchangeable salt–gel columns.

## Introduction

Many advanced techniques in molecular biology, genetics, forensics, molecular medicine, and biotechnology begin with the extraction of nucleic acids from biological samples. Examples of these techniques include nucleotide sequencing, DNA profiling, and nucleic acid–based methods for detecting pathogens and biological contaminants. Successful implementation of these techniques typically requires the removal of impurities that may cause artifacts, degrade or modify the target nucleic acid, or inhibit subsequent enzymatic reactions. However, the complete removal of impurities from the nucleic acid of interest can be challenging due to the complexity of the starting biological material. Such biological material often consists of a heterogeneous mixture of different types of molecules, such as proteins, polysaccharides, polyphenols, lipids, pigments, secondary metabolites, humic substances, small molecule enzyme inhibitors, peptides, and oligonucleotides. The main issue with current methods for purifying nucleic acids from complex biological mixtures is the low purity of the final sample or the low yield of the purified nucleic acid compared to the original amount in the starting sample. Therefore, although many DNA and RNA purification approaches have been described in the literature,^1−4^ there is a need in the art for a universal, rapid, and efficient method for isolating nucleic acids from even the most complex biological samples.

Previously, we reported a method for the efficient purification of HMW DNA for long-read sequencing^5^ that begins with electrophoresis in horizontal agarose gel-filled channels. After gel purification, DNA is electroeluted from the separating gel and trapped against a high-salt gel barrier. The method exploits the ability of excess counterions from the high-salt gel to reduce the electrophoretic mobility of DNA, causing it to accumulate in a buffer-filled gap between the separating gel and the high-salt gel. In this study, we present a significant improvement of this method, addressing the limitations of its previous version. We use vertical electrophoresis in two partially overlapping electrophoretic columns to rapidly extract target nucleic acids from complex biological samples with high yield and purity. The improved method is universally applicable to purifying HMW DNA and smaller DNA and RNA molecules. It is also easier to use, increasing its potential for scalability and automation. Finally, the vertical column method allows the sequential purification of several nucleic acid species from a single sample using interchangeable salt–gel columns.

## Experimental Section

### DNA Extraction Using the SDS/Proteinase K Method

We recommend using the SDS/Proteinase K method to prepare crude samples containing HMW DNA or total cellular RNA. Other methods frequently employ high concentrations of guanidinium chloride or guanidinium thiocyanate salts, necessitating additional salt removal steps. The following protocol can extract crude DNA or RNA for column loading from complex plant, animal, fungal, and microbial samples. The example below demonstrates how to extract crude nucleic acids from a 100 μg leaf sample.

1. Grind the deep-frozen leaf tissue in liquid nitrogen with a prechilled mortar and pestle. Transfer the ground powder to a 1.5 mL microtube.
2. Add 500 μL of lysis buffer (2% SDS, 10 mM EDTA, 50 mM Tris-HCl, pH 8.0) and proteinase K to a final concentration of 100–200 μg/mL. Mix and incubate at 55 °C for at least 1 h. Several hours to overnight incubation is preferred.
3. Add 85 μL of 10X loading buffer (20% (w/v) Ficoll 400, 100 mM Tris-HCl (pH 8.0), 5 mM EDTA, 0.01% Xylene Cyanol FF) to a final concentration of about 1.5X, mix, and store at 4 °C.

### HMW DNA Purification Protocol

Prepare a two-liter bottle of 1X THE running buffer^5^ by adding 40 mL of 50X THE buffer to 1960 mL of ultrapure water.

1. To cast the 0.8% separating gel (for the separating column), add 0.8 g of ultrapure agarose to 100 mL of 1X THE buffer in an Erlenmeyer flask. Swirl the flask gently to mix. Heat the flask with the agarose solution in a microwave oven at 800 W with occasional gentle swirling until all the agarose dissolves, forming a clear solution. Once the agarose solution has cooled to between 55 and 60 °C, carefully add about 1 mL to the bottom of the separating column (column diameter should not exceed 1.5 cm). The bottom of the column should be blocked with a porous membrane to prevent the gel from leaking out. Allow the gel to solidify and store at 4 °C until further use.
2. To cast the 0.8% high-salt gel, add 0.8 g of ultrapure agarose to 90 mL of 1X THE buffer in an Erlenmeyer flask. Swirl the flask gently to mix. Heat the flask with the agarose solution in a microwave oven at 800 W with occasional gentle swirling until all the agarose dissolves, forming a clear solution. Add 10 mL of 5 M NaCl to the solution, mix, and slowly cool at room temperature. Once the agarose solution has cooled to between 55 and 60 °C, carefully add about 2 mL to the bottom of the trapping column. The bottom of the column should be blocked with a porous membrane to prevent the gel from leaking out. Allow the gel to solidify and store at 4 °C until further use.
3. Fill the separating column completely with 1X THE buffer and place it vertically above an empty electrophoresis tank (e.g., using a chemistry stand with clamps). Fill the tank with 1X THE buffer and adjust the column’s position so that the bottom is only slightly covered. Avoid air bubbles.
4. Carefully load the sample (50–100 μL) onto the gel surface. Immerse a platinum electrode in the buffer above the gel and connect it to a power supply.
5. Run the gel at 100 V until the tracking dye (Xylene Cyanol FF) has just run out of the gel. Alternatively, DNA migration can be monitored during electrophoresis, and the run can be stopped when the DNA of interest has reached the end of the gel. Make sure that the DNA does not run out of the gel.
6. After turning off the power, disconnect the upper electrode, lift the clamp with the separating column, and attach the trapping column from below. The trapping column must be completely prefilled with 1X THE buffer. Avoid air bubbles.
7. Place the column assembly vertically above the electrophoresis tank (e.g., using a chemistry stand with clamps). Fill the tank with fresh 1X THE buffer and adjust the column assembly’s position so that the trapping column’s bottom is only slightly covered. Avoid air bubbles.
8. Return the platinum electrode to the buffer covering the separating column and connect it to a power supply.
9. Continue electrophoresis at 100 V for 5–10 min to elute the gel-purified DNA into the buffer-filled gap between the separating gel and the high-salt gel.
10. Turn off the power, carefully dismantle the column assembly without spilling the buffer, and use a pipet to collect the buffer containing the eluted DNA from the trapping column.

### Control of DNA Quality and Integrity

The ratio of absorbance at 260 and 230 nm (A_260_/A_230_) was used to estimate DNA purity. The absorbance values were measured using a NanoDrop ND-2000 spectrophotometer (Thermo Fisher Scientific). HMW DNA concentrations determined with NanoDrop were validated with a Qubit 3.0 fluorometer (Invitrogen) using the dsDNA BR assay kit (Thermo Fisher Scientific). DNA integrity was assessed by electrophoresis on 0.8% agarose gels.

### Long-Read Sequencing with Oxford Nanopore Technologies (ONT)

Sequencing libraries were prepared using the Rapid Barcoding Kit (SQK-RBK110.96, Oxford Nanopore Technologies) and Solid-Phase Reversible Immobilization (SPRI) beads for DNA cleanup following the manufacturer’s instructions. Duplicate runs with different barcodes were performed for each sample, and a single sample was pooled for sequencing on a single flow cell. Each library containing ∼0.5 μg of DNA was sequenced on the ONT GridION platform using R9.4.1 chemistry. Sequencing ran for 25 h until the flow cell buffer was exhausted. Data acquisition, real-time analysis, and sample tracking were performed using MinKNOW (v. 2.1) software. High accuracy base calling was performed from the fast5 files using the Oxford Nanopore Guppy tool (v. 3.0.4). The run was monitored using RAMPART (https://github.com/artic-network/rampart), which allowed the run to be stopped when a minimum sequencing depth of 20x was reached.

## Results and Discussion

### Method Implementation

The principle of the method was described previously.^5^ Briefly, the release of cations (counterions) from a gel containing a high concentration of salt electrostatically shields a nucleic acid’s negative charge, reducing its electrophoretic mobility.^6,7^ Consequently, the high-salt gel may act as an electrophoretic trap for electroeluted nucleic acids. In the original implementation of the method, we employed nucleic acid electrophoresis in a horizontal channel filled with agarose gel. Two gel sections were excised downstream of the loading well, forming a sample collection reservoir and a mold cavity for the high-salt gel. The mold cavity was then filled with a molten agarose solution with a high concentration of salt. After the high-salt gel had solidified, it was removed from the channel and stored for later use during electroelution. Horizontal gel electrophoresis was next performed to separate the HMW DNA of interest from impurities. When the HMW DNA reached the end of the separating gel, the high-salt gel block was returned to its original position. The purified DNA was then electroeluted into the gap between the separating gel and the high-salt gel, where it accumulated due to the decrease in electrophoretic mobility. In the final step, the HMW DNA was collected from the gap, which we designated the sample collection reservoir. It is apparent that, although this method effectively isolates HMW DNA with high yield and purity from complex biological samples, it is still labor-intensive and involves multiple steps.

**Figure 1:**
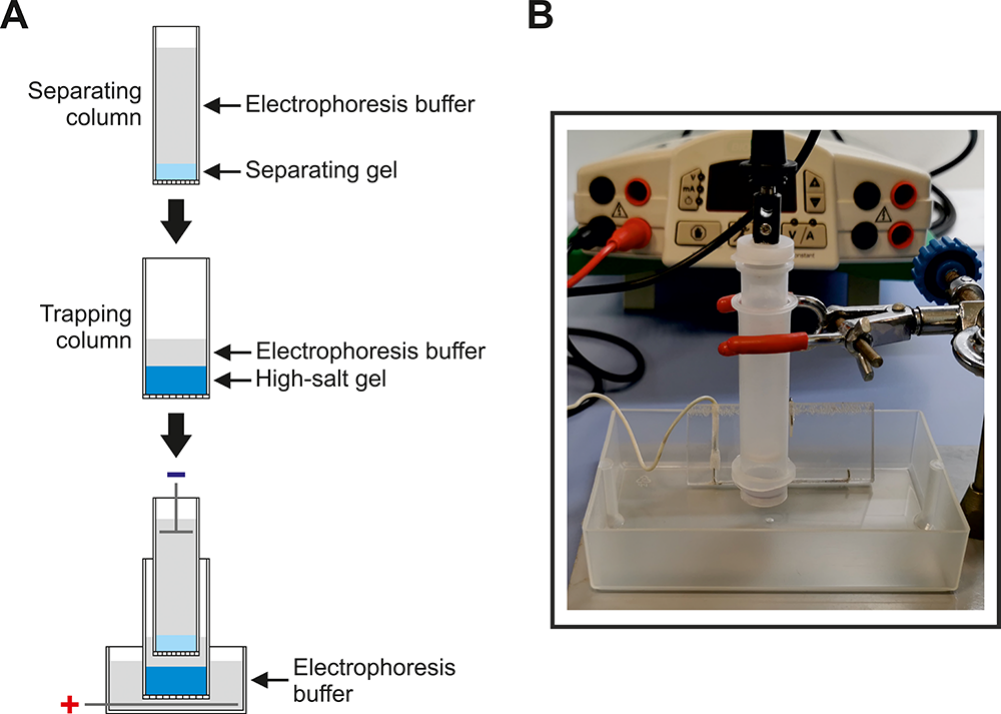
An improved device for nucleic acid purification based on vertical column electrophoresis. (A) Schematic of the device consisting of two nested electrophoretic columns. The upper column is used to purify target nucleic acids by electrophoresis in a thin layer of precast separating gel. The lower column contains a precast high-salt gel at the bottom overlaid with electrophoresis buffer. (B) Photograph of the prototype device.

The current study aimed to make the method more user-friendly, efficient, and suitable for automation and scalability. In the new version of the method, we switched from horizontal to vertical electrophoresis. The method employs two nested, partially overlapping electrophoretic columns (Figure 1A and 1B).

**Figure 2:**
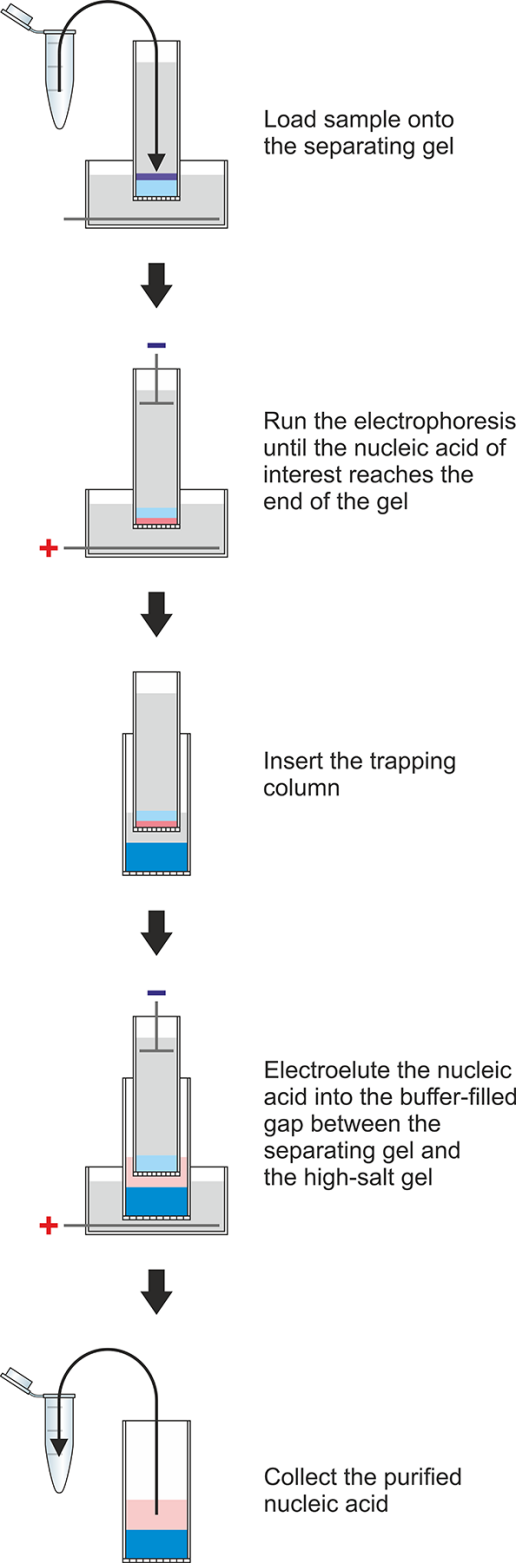
Schematic representation of the nucleic acid purification workflow.

The upper column, which is smaller in diameter (1–1.5 cm), has a thin layer of agarose gel at the bottom covered with electrophoresis buffer. The purpose of this column is to separate target nucleic acids from impurities in a single step. A crude sample containing the nucleic acid of interest (e.g., an SDS/Proteinase K- extracted sample) is loaded onto the surface of the gel in the upper column and subjected to electrophoresis (Figure 2). When the nucleic acid reaches the end of the gel, the power is temporarily turned off, and a second, larger-diameter column is inserted from below. This column, which contains a layer of high-salt gel overlaid with electrophoresis buffer, is inserted on the outside of the upper column so that the two columns partially overlap. The purpose of the lower column is to serve as a nucleic acid trap. When the power is restored, the purified nucleic acid is electroeluted from the upper separating gel into the buffer-filled gap between the separating gel and the high-salt gel. Excess counterions from the high-salt gel slow its migration and cause it to accumulate. Finally, the power is disconnected and the nucleic acid solution in electrophoresis buffer is collected from the top of the trapping column.

In addition to purifying individual nucleic acid molecules of interest, the improved method can be used to purify total nucleic acids from difficult-to-separate impurities like polysaccharides. In this case, the lower trapping column should be inserted into the upper column within a few minutes of starting electrophoresis. By this time, the small, charged impurities will have migrated out of the separating gel. The purified total nucleic acids are then electroeluted into the buffer above the high-salt gel, while uncharged or low-charged impurities, such as polysaccharides, remain trapped in the upper column.

### Advantages of the Improved Method

**Figure 3:**
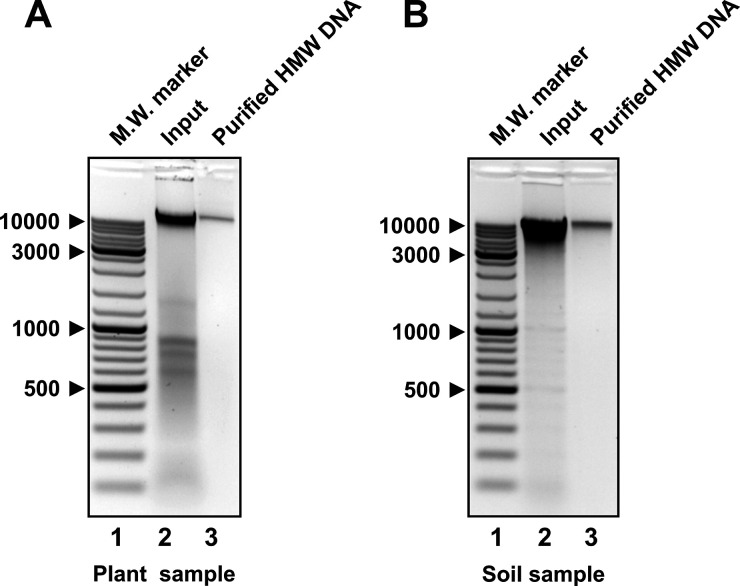
Proposed method yields high-purity HMW DNA from complex plant and soil samples. Negative images of ethidium bromide-stained agarose gels are shown. (A) Lane 1: molecular weight marker (GeneRuler DNA ladder, Thermo Fisher Scientific). Lane 2: crude nucleic acid preparation extracted with SDS/Proteinase K from *Zingeria trichopoda* leaves, which served as an input for HMW DNA purification using the proposed method. Lane 3: purified HMW DNA. (B) Same as (A) except that crude, CTAB-extracted DNA from a soil sample was used as an input for HMW DNA purification. Molecular weight marker sizes are shown in base pairs.

The first and most obvious advantage of the proposed method over its predecessor is its simplicity and ease of use. There is no longer a need for delicate manipulations such as gel excision, filling the excised cavity with molten high-salt agarose, gel removal, and subsequent reinsertion. Since both gels in the upper and lower electrophoretic columns are precast, user handling of the gels is no longer necessary. Vertical electrophoresis also eliminates the need to precisely load samples into loading wells. As a result, the improved method is just as effective as its predecessor in purifying target nucleic acids from complex biological mixtures (Figure 3), but it is more efficient and user-friendly.

**Figure 4:**
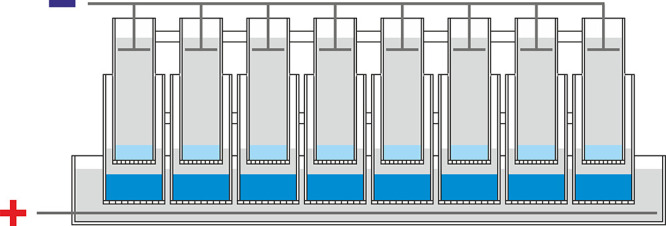
Schematic of a high-throughput device featuring multiple electrophoretic columns operating simultaneously.

Because the method is simpler, robotics can more easily automate repetitive tasks such as sample loading, trapping column insertion, and sample collection. The method’s simplicity also enhances its scalability by allowing multiple columns to be run in parallel for high-throughput tasks (Figure 4).

Another advantage of the improved method over the previously published horizontal procedure is more efficient sample collection. In the original method, the purified DNA sample is collected from the buffer-filled reservoir located between the separating gel and the inserted high-salt gel. However, when the high-salt gel is inserted into an electrophoretic channel, a perfect seal cannot be achieved. As a result, some current will bypass the gel, causing sample loss during electroelution. More importantly, the current will also pass through the electrophoresis buffer above the submerged gel, partially washing out the electroeluted nucleic acid and reducing the yield. This issue is inherent in horizontal electrophoresis, where gels must be fully submerged in an electrophoresis buffer^8^ to prevent band distortion and reduce heating. The improved method, based on vertical column electrophoresis, is free of these limitations. First, there is a tight seal between the precast high-salt gel and the column wall; second, the current cannot bypass the trapping column. This results in higher yields and significantly faster sample processing, which is especially important for high-throughput applications. In addition, vertical electrophoresis eliminates the problem encountered with horizontal electrophoresis, where some sample is inevitably lost into the buffer during and shortly after loading into the well.

The previously published method focused on isolating HMW DNA primarily for long-read nucleotide sequencing.^5^ Because it required several gel handling steps, it was not well suited for purifying multiple nucleic acid species from the same sample. In contrast, the improved method based on vertical column electrophoresis provides a simple and robust solution for such tasks. By using interchangeable lower trapping columns, several species of purified nucleic acids, if sufficiently different in size, can be collected one at a time as they reach the end of the separating gel in the upper column. For example, total RNA can be collected separately from HMW genomic DNA. Thus, an advantage of the improved method is that it can be universally applied to isolate DNA or RNA molecules of different sizes from a single complex biological sample.

Another important difference between the original horizontal design and the vertical column-based design proposed here is that the latter makes it technically more feasible to add a UV absorbance detector to track nucleic acid migration in real time. Thus, the proposed column-based method has an additional advantage in nucleic acid detection, allowing for proper timing of the electroelution trap insertion.

## Conclusions

This study presents an improved method and device for isolating nucleic acids, including high-molecular-weight DNA, from a wide range of biological samples such as blood, soil, herbarium, feces, and tissues rich in polysaccharides, polyphenols, pigments, and secondary metabolites. The method can become a valuable addition to the arsenal of nucleic acid purification methods for various demanding applications, including long-read nucleotide sequencing. Like its predecessor, the improved method purifies nucleic acids of interest in a single step from complex biological samples containing large amounts of chemically diverse impurities but only small amounts of target nucleic acid. However, the improved method outperforms the original method in several ways. Eliminating gel handling has made the technique more efficient and user-friendly, allowing for greater automation and scalability. Furthermore, the switch from horizontal to vertical electrophoresis has increased the method’s speed while maintaining high target nucleic acid yield and purity. Finally, the method has become more universal, allowing sequential purification of different-sized DNA or RNA species from the same sample using interchangeable salt–gel columns.

## Data Availability

The original gel electrophoresis data of this study are publicly available in Figshare at 10.6084/m9.figshare.25441618.v1, and the original ONT data reports are openly available in Figshare at 10.6084/m9.figshare.25998718.v1.

## Supporting Information Available

Long-read sequencing report with Oxford Nanopore Technologies of different soil samples using different DNA extraction methods and ONT protocol for GridION (PDF)
