# Supplementary material for: An Improved Method and Device for Nucleic Acid Isolation Using a High-Salt Gel Electroelution Trap
Source: Anal Chem. 2024 Sep 17;96(39):15526–30. doi: 10.1021/acs.analchem.4c03720 (PMC11447668; doi:10.1021/acs.analchem.4c03720)
Supplement: Supplementary file 1 — ac4c03720_si_001.pdf [file ac4c03720_si_001.pdf]

Supporting Information

An Improved Method and Device for Nucleic Acid Isolation Using a High-Salt Gel Electroelution Trap

Ruslan Kalendar [a]*, Konstantin I. Ivanov [b,c]*, Ilyas Akhmetollayev [d], Ulykbek Kairov [a], Olga Samuilova [e,f], Timo Burster [g], Andrey A. Zamyatnin Jr. [e,h,i]

[a] National Laboratory Astana, Nazarbayev University, 53 Kabanbay batyr Ave., Astana, 010000, Kazakhstan

[b] Department of Microbiology, Viikinkaari 9, University of Helsinki, Helsinki, 00014, Finland

[c] Research Center for Translational Medicine, Sirius University of Science and Technology, Olympic Ave., 1, Sochi, 354340, Russian Federation

[d] National Center for Biotechnology, 13/5, Kurgalzhynskoye road, Astana, 010000, Kazakhstan

[e] Department of Biochemistry, Sechenov First Moscow State Medical University, 119991 Moscow, Russian Federation

[f] HSE University, 10100 Moscow, Russian Federation

[g] Department of Biology, School of Sciences and Humanities, Nazarbayev University, Kabanbay Batyr Ave. 53, Astana, 010000, Kazakhstan

[h] Faculty of Bioengineering and Bioinformatics, Lomonosov Moscow State University, 119234 Moscow, Russian Federation

[i] Belozersky Institute of Physico-Chemical Biology, Lomonosov Moscow State University, Moscow, 119234, Russian Federation

*Corresponding authors:

Ruslan Kalendar, ruslan.kalendar@helsinki.fi, phone: +358504483505

Konstantin I. Ivanov, konstantin.i.ivanov@gmail.com, konstantin.ivanov@helsinki.fi

R.K.: ruslan.kalendar@helsinki.fi ORCID 0000-0003-3986-2460

K.I.: konstantin.i.ivanov@gmail.com ORCID 0000-0001-9198-5674

I.A.: iliyas@mail.ru ORCID 0000-0002-6219-4002

U.K.: ulykbek.kairov@nu.edu.kz ORCID 0000-0001-8511-8064

O.S.: olga.v.samuilova@gmail.com ORCID 0000-0002-1195-9116

T.B.: timo.burster@nu.edu.kz ORCID 0000-0002-9596-6558

A.Z.: zamyat@belozersky.msu.ru ORCID 0000-0002-3046-4565

Table S1. Long-read sequencing report with Oxford Nanopore Technologies of different soil samples using different DNA extraction methods.

**Flow cell ID**	**Number of pores**	**Number of samples**	**Sample ID**	**Barcode ID**	**OUTLIERS Read length (kb) Outliers'?**	**Read length (kb)**	**Estimated reads**	**Base-called reads**
FAS63057 (X1)	922	1	1 soil DNA purified with gel	1	27.75 - 28	2.78 k	5.55 Mb	4.78 Mb
FAS47629 (X2)	1477	2	2 soil DNA purified with gel	2	114 - 115	14.16 k	43.65 Mb	38.56 Mb
			1_2 soil DNA purified with gel	3				
FAV38481 (X3)	961	3	2_2 soil DNA purified with gel	4	63 - 63.5	8.74 k	22.43 Mb	20.15 Mb
			3_2 soil DNA purified with gel	5				
			4_2 soil DNA purified with gel	6				
FAU49011 (X5)	1464	2	21 soil DNA purified with the E.Z.N.A. soil DNA Extraction Kit	7	114 - 115	2.4 k	7.97 Mb	6.59 Mb
			36 soil DNA purified with the E.Z.N.A. soil DNA Extraction Kit	8				

ONT protocol for GridION (07.06.2023)

Before start Qubit conc= 17,0 ng/µl.

Total number of samples- 10(soil DNA). Barcodes from 1 to 10.

1. From each sample, take 10 µl.
2. Add 5 µl of Rapid Barcode Plate for each.
3. Incubate at 30°C for 2 min, then 80°C for 2 min
4. Transfer all samples to 1,5 ml Eppendorf DNA LoBind tube.
5. Resuspend SPRI beads.
6. Add an equal volume of resuspended SPRI.
7. Incubate on a Hula mixer (rotator mixer) for 10 min.
8. Prepare 1ml of fresh 80% ethanol in nuclease-free water.
9. Spin down the sample and pellet on a magnet. Keep the tube on the magnet, and pipette off the supernatant.
10. Keep the tube on the magnet and wash the beads with 1ml of freshly prepared 80% ethanol without disturbing the pellet. Remove the ethanol using a pipette and discard.
11. Repeat previous step.
12. Briefly spin down and place the tube back on the magnet. Pipette off any residual ethanol. Allow drying for 30 seconds, but do not dry pellet to the point of cracking.
13. Remove the tube from the magnetic rack and resuspend the pellet by pipetting in 15 µl Elution Buffer (EB). Incubate for 10 minutes at a room temperature.
14. Pellet the beads on a magnet until the eluate is clear and colorless.
15. Remove and retain 15 µl of eluate containing the DNA library into a clean 1,5 Eppendorf DNA LoBind tube *Quantify DNA concentration by using Qubit ds HS Assay kit.*
16. QUBIT Conc. after pooling and clean-up: 77,6 ng/ µl
17. Add 1 µl of Rapid Adapter F (RAP F)
18. Incubate at room temperature for 5 min.

LOADING

Flow cell name FAU49013

Pores 1529

Position x1

Time 25 hours

Total loading mix- 94 µl (SBII-47 µl; LBII- 32 µl; Library- 15 µl)
